# Closure of the appendiceal stump in laparoscopic appendectomy: A systematic review of the literature

**DOI:** 10.1016/j.amsu.2020.07.058

**Published:** 2020-08-04

**Authors:** N. Makaram, S.R. Knight, A. Ibrahim, P. Patil, M.S.J. Wilson

**Affiliations:** a Department of General Surgery, Ninewells Hospital and Medical School, Dundee, DD1 9SY, UK; b Usher Institute, University of Edinburgh, Edinburgh, EH16 4XU, UK; c Department of General Surgery, Forth Valley Royal Hospital, Larbert, FK5 4WR, UK

**Keywords:** Appendicitis, Appendicectomy, Stump closure, Review, EL, Endoloop, LA, Laparoscopic Appendicectomy

## Abstract

**Background:**

Closure of the appendiceal stump is a key step performed during laparoscopic appendicectomy. Inadequate management of the appendiceal stump has the potential to cause significant morbidity. Several methods of stump closure have been described, however high-level evidence is limited. We performed a systematic review evaluating clinical outcomes and quality of the evidence for the methods of appendiceal stump closure.

**Methods:**

A systematic literature search was performed using Medline, Embase, Cochrane Database and Google Scholar to identify studies comparing appendiceal stump closure methods in laparoscopic appendectomy for acute appendicitis from inception to October 2019. Data regarding operative duration, peri-operative complications, length of stay and costs were collated from all included studies.

**Results:**

From 160 identified studies, 19 met the inclusion criteria. Endoloops and endoclips provide equivalent clinical outcomes at lower cost, while operative duration was shortest with endoclip closure. Endostapler devices have the lowest rate of peri-operative complications (3.56%), however their cost limits their regular use in many healthcare environments. Post-operative complication rate and length of stay were similar for all stump closure methods. Conclusion: Although there are no significant differences in method of stump closure in laparoscopic appendectomy, closure with endoclips provides the shortest operative duration. There is a need for robust and standardized reporting of cost data when comparing stump closure methods, together with higher level evidence in the form of multi-centre randomized controlled trials before firm conclusions can be drawn regarding the optimal method of stump closure.

## Highlights

• Currently there is no robust higher order evidence which assesses clinical outcomes, cost and time efficiency of methods of stump closure in laparoscopic appendicectomy.
• This is the first systematic review to investigate clinical outcomes, cost and quality of current evidence for all methods of laparoscopic appendiceal stump closure in acute appendicitis.
• No difference was observed in methods of stump closure in complication rate, length of stay, cost, and time efficiency.
• Endostaplers appear to provide most robust closure, albeit at a higher economic cost.
• Endoloops appear to provide efficient and successful closure, and are currently the most frequently studied method of closure.

## 1 Introduction

Acute appendicitis is one of the most common surgical emergencies requiring acute hospital admission. In the UK, acute appendicitis has an incidence of 52 per 100,000 [1] and is the most common presentation of the acute abdomen [2].

The majority of appendectomies are undertaken laparoscopically, with evidence demonstrating reduced post-operative pain, a lower rate of wound infection, faster recovery and shorter hospital stay when compared with the open procedure [3,4]. However, the laparoscopic approach is associated with longer procedure times, increased cost and a higher risk of post-operative intra-abdominal abscess formation [[5], [6], [7]]. Furthermore, appendiceal stump leakage secondary to inadequate stump closure is a recognized complication following laparoscopic appendectomy (LA) [8].

Multiple methods of laparoscopic closure of the appendiceal stump have previously been described, including; endoloops (EL), endoclips (both metallic and polymeric clips), linear stapler devices, suture ligation and endocoagulation [9,10]. However, there is no consensus as to the optimal method of appendiceal stump closure. Current literature suggests the relative advantages and disadvantages of such methods in securing the appendiceal stump but to our knowledge a systematic review of the current evidence is yet to be performed.

A recent United Kingdom (UK) nationwide survey which assessed the current UK practice for securing the appendiceal stump, showed EL to be overwhelmingly the preferred method (86.5%) [11]. The most influential factors for the method of choice for securing the stump were ease of application and severity of stump inflammation. 34.2% of respondents noted that cost was a major influence of method of choice.

Our aim was to perform a systematic review analyzing the current evidence for each method of appendiceal stump closure during LA in acute appendicitis, identify potential gaps within the literature and attempt to determine future research priorities.

## 2 Materials and methods

The systematic review was performed in concordance with the PRISMA guidelines [12]. Institutional Review Board approval was not required. Written consent was not required given the nature of the systematic review.Medline, Embase and Cochrane databases were searched between January 1974 to November 2019, with limits of English Language and full text articles, using the MeSH search terms: ‘Appendicitis OR appendicectomy OR appendix’ (6395) AND ‘Laparoscopic OR laparoscopically OR minimally invasive’ (28,407) AND ‘Stump closure OR ligation OR closure OR stapler OR stapling OR endostapler OR linear stapler OR endoloop OR suture OR hem-o-lok OR polymeric clip OR clip’ (80,129). Inclusion criteria were any study that examined one or more methods of appendiceal stump closure during LA for acute appendicitis. Articles where subjects were <18 years old, case reports, conference proceedings or editorials were excluded. The age restriction was to ensure the exclusion of a paediatric population such that the population studied was homogeneous. This review is registered with PROSPERO (CRD42019153567).

All abstracts identified in the initial search were assessed independently by two individuals (NM and SK) to determine which studies met the inclusion criteria, with disagreements resolved by discussion. Further relevant articles were identified by manual screening of included article reference lists and the performance of grey literature searches through Google scholar.

For each included study data were collected for operative time, peri-operative complications, length of in-patient stay and cost for stump closure method used, if available. To enable comparison of costs between studies, each figure was converted to an equivalent amount in Euros using the average conversion rate for the year of publication [13].

For operative length, duration of in-patient stay, complication rates and associated costs, means were calculated across studies to enable direct comparison between different methods of stump closure. Analysis of differences between methods of stump closure were performed using ANOVA with use of a post-hoc Tukey analysis. All statistical analysis was performed using the R statistical package [14] with the P value set at <0.05 for statistical significance.

## 3 Results

**Fig. 1:**
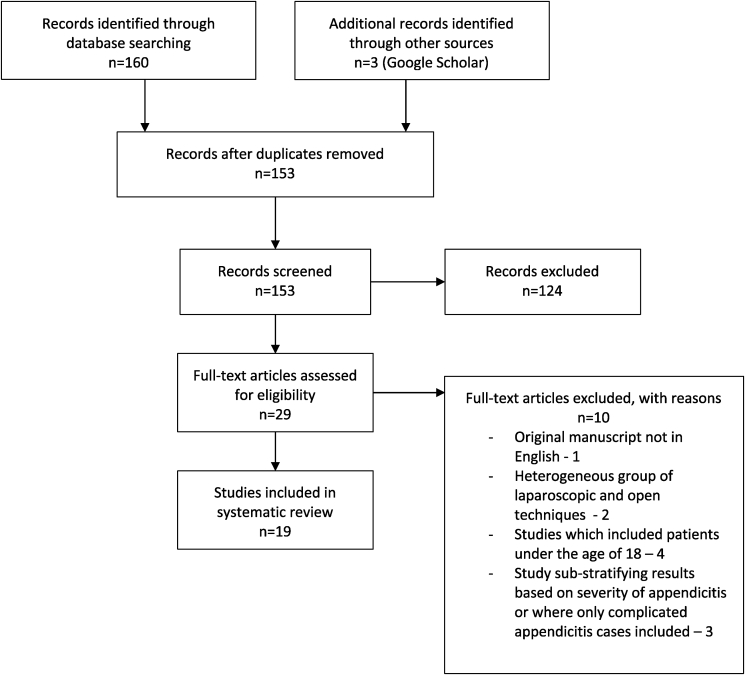
Literature search PRISMA flow diagram.

160 studies were identified from the initial search, and after duplicates were removed 153 studies were suitable. Following manual screening, 19 were deemed suitable for inclusion in the systematic review (Fig. 1). Eleven studies were prospective, of which three were randomized controlled trials. The remaining eight studies were retrospective in nature (Table 1). The average number of patients per study was 562, with a range of 28 to 5846 patients.

**Table 1:** Summary of studies included in the systematic review.

Author (year)	Study design	Sample number (n)	Comparitors	Average Age (years)	Perioperative Complication Rate (%)	Postoperative complication Rate (%)	Overall Complication rate (%)	Average Length of Stay (days)	Average Operative Duration (minutes)	Cost (euros)
**Endoloops**										
Beldi (2006)	Prospective	2565	EL vs Staple	28	1.10%	5.30%	6.40%	5.4	53.4	16
Rakic (2014)	Prospective	163	EL vs Staple	26	0.61%	4.90%	5.51%	4.0	48	554.9
Swank (2014)	Retrospective	571	EL vs Staple	34 (med)	6.70%	9.80%	16.50%	2	60	n/a
Sahm (2010)	Prospective	1135	EL with selective staple	32	4.32%	1.94%	6.26%	n/a	47.33	n/a
Kiudelis (2013)	Prospective	112	EL vs suture	32.4	3.60%	6.30%	9.90%	2.4	58.4	460
Delibegovic (2009)	Prospective	24	EL vs Polymeric clip	28.7	0.00%	0	0.00%	2.2	47.1	88.5
Colak (2013)	RCT	27	EL vs Polymeric clip	26.8	0	11.1%	11.10%	2.5	75.4	35.12
Jenwitheesuk (2012)	Retrospective	23	EL vs Polymeric clip	26	3.85%	0	3.85%	3.17	66	n/a
Lucchi (2017)	Retrospective	121	EL vs Polymeric clip	29.9	0	1.65%	1.65%	1.2	40.5	92
Wilson (2018)	Retrospective	78	EL vs polymeric clip	28.2	0	5.1%	5.1%	2.9	68	24.36
Kim (2018)	Retrospective	75	EL vs Staple	38.3	0	18.6%	18.6%	0.7	38.5	1680
**Staple**										
Beldi (2006)	Prospective	3281	Staple vs EL	30	1.30%	5.90%	7.20%	5.9	51.7	306
Rakic (2014)	Prospective	75	Staple vs EL	38	1.33%	9.33%	10.66%	3.6	55	970.7
Swank (2014)	Retrospective	465	Staple vs EL	36	10%	8.60%	18.60%	2	58	n/a
Sahm (2010)	Prospective	43	Selective staple vs EL	46	2.32%	6.98%	9.30%	n/a	76.6	n/a
Hanssen (2007)	Prospective	14	Staple vs Polymeric clip	n/a	0	0	0.00%	2.78	62.4	264.92
Al-Temimi (2017)	Prospective	47	Staple vs Polymeric clip	32.1	6.38%	19.1%	25.5%	2.5	38.8	259.64
Kim (2018)	Retrospective	250	Staple vs EL	38.2	0	24.0%	24.0%	1.1	42.4	2005
Kliuchanok (2019)	Retrospective	410	Staple vs Polymeric clip	41.7	2.2%	7.6%	9.8%	3.7	55.3	348.7
**Clip**										
Colak (2013)	RCT	26	Polymeric clip vs EL	31.9	0	11.50%	11.5%	2.1	64.7	8.78
Delibegovic (2009)	Prospective	28	Polymeric clip vs EL	26.6	3.57%	0%	3.6%	2.2	38.7	76.9
Hanssen (2007)	Prospective	14	Polymeric clip vs Staple	n/a	0.00%	0%	0.0%	2	53.4	192.82
Jenwitheesuk (2012)	Retrospective	68	Polymeric clip vs EL	32	2.94%	0%	2.9%	2.5	38	n/a
Strzalka (2014)	Retrospective	93	Metal clips	33.6	0.00%	7%	7.0%	3.38	66	n/a
Gonenc (2012)	RCT	61	Metal clip vs suture	26.76	1.60%	4.8%	6.4%	0.796	46.3	n/a
Rickert (2012)	Prospective	100	Metal clip	30.6	0.00%	3%	3.0%	4	54	n/a
Alis (2012)	Retrospective	233	Metal clip	28.4	3.00%	5%	8.0%	0.75	31.1	3.50
Ates (2012)	RCT	30	Metal clip vs suture	28.23	20.0%	3%	23.0%	2.07	41.3	n/a
Al-Temimi (2017)	Prospective	45	Polymeric clip vs Staple	27.97	11.10%	2.20%	13.30%	1.8	43.3	29.38
Lucchi (2017)	Retrospective	138	Polymeric clip vs EL	32.8	0	2.17%	2.17%	1.23	36.4	48
Wilson (2018)	Retrospective	47	Polymeric clip vs EL	32.1	0	4.26%	4.26%	3.2	59.0	24.36
Kliuchanok (2019)	Retrospective	208	Polymeric clip vs Staple	33.6	1.0%	1.4%	2.4%	2.9	51.0	19.94
**Suture**										
Kiudelis (2013)	Prospective	40	Suture vs EL	32.1	2.50%	5%	7.5%	2.8	79.6	n/a
Ates (2012)	RCT	31	Suture vs Metal Clip	29.35	13%	10%	23.0%	2.06	62.81	n/a
Gonenc (2012)	RCT	46	Suture vs Metal Clip	27.4	4.20%	8.50%	12.70%	0.846	61.9	n/a

## 4 Endoloops

Eleven studies assessed the effectiveness of endoloops in securing the appendiceal stump, totaling 4894 patients (Table 1) [[15], [16], [17], [18], [19], [20], [21], [22], [23], [24], [25]]. The majority of studies collected data prospectively, with five retrospective in nature [17,[22], [23], [24], [25]]. The overall mean patient age was 30 years. Operative time averaged 54.8 min (range 47–66) (Table 2). The overall complication rate was 7.69%, with two-thirds of these complications occurring post-operatively (4.61%). Length of stay was measured in ten studies with a mean of 2.7 days (range 2–5.4). Costs were measured by a variety of means, including per loop and per operation (Table 1).

**Table 2:** Mean perioperative and postoperative complication rates of each method of closure relating to average operative time, age and length of stay of subjects assessed.

Device	Subjects (n)	Mean Age (years)	Average Operative Time (minutes)	Perioperative Complication Rate (%)	Postoperative Complication Rate (%)	Average length of stay (days)	
**Clip**	1091	30.09	47.7	3.32	3.55	2.17	
**Endoloop**	4894	30.05	54.8	2.52	4.61	2.65	
**Suture**	117	29.50	68.2	6.57	7.83	1.84	
**Staple**	4585	36.70	55.0	3.56	8.34	2.98

**Table 3:** a and b. Specified Complications and their respective overall average incidence for each method of appendiceal stump closure.

a. Perioperative complications				
Device	Intraop/Postop Bleeding/Haematoma (%)	Access related (plus requiring conversion) (%)	Organ Lesion/injury (%)	Slipped Clip (only applicable to clips) (%)
**Polymeric Clip**	0.89	0.00	0.00	0.00
**Metallic Clip**	0.74	0.00	0.85	0.85
**Endoloop**	1.41	0.11	0.30	-
**Suture**	0.83	2.92	0.70	-
**Staple**	0.44	0.02	0.38	-

b. Postoperative complications								
Device	Readmission/Reoperation (%)	Small Bowel Obstruction (%)	Peritonitis (%)	Nonsurgical/other (%)	Stump leak (%)	ICU stay (%)	Superficial surgical site Infection (%)	Abscess formation (%)
**Polymeric Clip**	0.00	0.00	0.00	0.95	0.00	0.00	2.29	0.37
**Metallic Clip**	0.22	1.12	0.00	0.44	0.44	0.00	0.72	1.37
**Endoloop**	0.29	1.21	0.02	0.46	0.00	0.15	2.15	2.02
**Suture**	0.00	1.43	0.70	3.33	0.00	0.00	1.81	1.67
**Staple**	1.35	0.94	0.00	0.00	0.00	0.24	1.02	2.05

## 5 Endoclips

Thirteen studies assessed closure of the appendiceal stump using endoclips, totaling 1091 patients (Table 1) [[20], [21], [22], [23], [24],[26], [27], [28], [29], [30], [31], [32], [33]]. Of these, three were randomized controlled trials, four were prospective observational studies, and six were retrospective studies. Two different types of clip were investigated - metallic clips in five studies and polymeric clips in the remaining eight. The mean age of patients was 30.1 years. One study failed to report patient age [26]. Mean operative duration was 47.7 (range 31.1–66) minutes. Length of stay was 2.2 days (range 0.8–4.0 days, Table 2). The overall complication rate associated with endoclips was 7.1%. Metallic clips produced a mean complication rate of 9.5%, and polymeric clips produced a mean complication rate of 5.4%. Upon statistical analysis using a two-sided Wilcoxon rank sum test, neither the perioperative complication rate (p = 1), polymeric clips 1.76%, metal clips 2.7%) nor the postoperative complication rate (p = 0.1019, polymeric clips 2.07%, metal clips 4.83%) was found to be significantly different between metallic clips and polymeric clips.

Cost was evaluated in eight studies, with methods varying between evaluating cost per clip, per pack of clips (with varying numbers of clips included per pack and often not specified) and per procedure. Hanssen et al. [26] estimated cost per operation at €192.82, however this cost was substantially reduced in other studies [20,21,30]. Lucchi et al. [23], Wilson et al. [24] and Al-Temini et al. [32] all estimated costs per pack. The average cost per clip across studies was €29.73 and €33.40 per pack.(see Table 3)

## 6 Endostapler devices

The use of an endostapler device to secure the appendiceal stump was assessed in eight studies involving a total of 4585 patients. Five were prospective studies [15,16,18,26,32], while the remaining three were retrospective [17,25,33]. The mean age of patients was 36.7 years (Table 2). One study failed to report patient age [26]. Operative duration averaged 55.0 min, while length of stay was measured in seven studies and averaged 3 days (range 2–5.9). The overall complication rate was 13.6%. Cost was estimated in five studies, all by operation. This cost averaged €692.49 (range €240.78 to €2005).

## 7 Suture closure

Three studies evaluated the use of laparoscopic sutures to secure the appendiceal stump with all studies prospectively designed [19,28,31]. Two studies were randomized control trials [28,31]. 117 patients were included (Table 1) with a mean age of 29.6 years and an operative duration of 68.2 min (range 61.9–79.6, Table 2). Mean length of stay was 2.2 days (range 0.846–2.8) while the overall complication rate was 14.4%, thus the highest of all methods of closure.

Cost was not reported in any of the studies, but was discussed in general terms. There was consensus between the studies, which highlighted the reduction in equipment-related cost when securing the appendiceal stump with instrument-tied sutures, however the longer operating time was likely to offset any savings gained.

## 8 Statistical analysis of differences between methods of closure

**Fig. 2:**
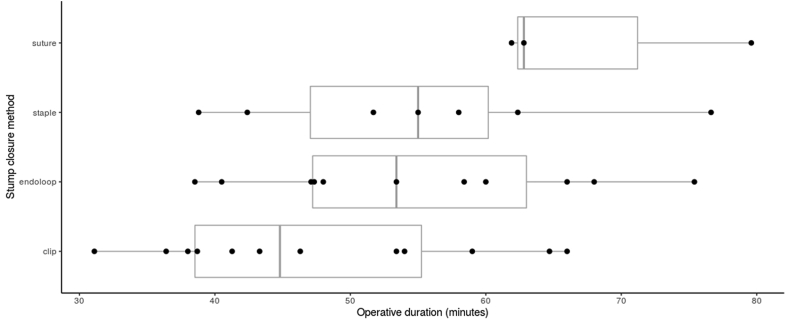
Operative duration of each method of closure of the appendiceal stump.

There were differences in operative duration between methods of closure employed, although these were not statistically significant (p = 0.0716, F statistic 2.59, Endoloops = 54.8, Clip = 47.7, Suture 68.2, Staple = 55.0). Clips provided the shortest operative duration. Due to the variability observed in the data recorded by the various studies, the significance of the method of stump closure in influencing operative duration is difficult to assess. The distribution of operative duration in each method of stump closure is illustrated in Fig. 2.

There was no significant difference observed between methods of stump closure in each of perioperative complication rate (p = 0.670, F-statistic 0.525, Clip 3.32%, endoloops 2.52%, Suture 6.57%, Staple 3.56%), postoperative complication rate (p = 0.103 F-statistic 2.27, clip 3.55%, endoloop 4.61%, suture 7.83%, staple 8.34%), length of stay (p = 0.493, F-statistic 0.823, clip 2.17 days, endoloop 2.65 days, suture 1.84 days, staple 2.98 days), and cost (p = 0.0949, F-statistic 2.71).

## 9 Discussion

To our knowledge this is the first systematic review that has investigated the clinical outcomes and quality of the current evidence for all methods of laparoscopic appendiceal stump closure in acute appendicitis. We report no statistically significant difference in complication rate, length of stay, or cost between methods of stump closure. Endoclips provided the most time-efficient method of closure, but this did not reach statistical significance. Suture closure, although the cheapest method, has a high complication rate and current evidence suggests this method should be avoided.

Endostaplers appear to be among the most robust closure methods, however they appear to also be associated with high postoperative complication rates, and the associated costs limit its use in all but the most severe cases of appendicitis. It may well be that the selective use of endostaplers in the most severe cases of appendicitis accounts for their perceived high postoperative complications rates through selection bias.

EL were the most frequently studied method of stump closure, with over 4500 patients across all studies. The majority were well-designed prospective studies in which EL were compared with polymeric clips. The rate of intraoperative complications was low at 2.5%. The current literature suggests EL provide an efficient and easy to perform technique for closure, with low risk of intraoperative complications [34]. However post-operative complication rate was 4.61%. It has been hypothesized that there is an increased risk of abscess formation secondary to exposure of contaminated mucosa following the use of endoloops [30,35].

Beldi et al. [15] published a large prospective series that supports the increased post-operative complication rate observed in the use of EL when compared with staples, although the difference was not statistically significant. On the other hand, Swank et al. [17] report no significant difference in operative time or peri-operative complications when comparing endostapler to EL. They concluded that EL were the better option as they gave similar operative time and complication rates compared with endostapler, but at a greatly reduced cost. However, complications may have been under-reported due to the retrospective nature of the studies. Indeed, Sahm et al. [18] observed a lower incidence of intra-abdominal abscess with EL compared to staple, together with a shorter operative time by 19 min in their retrospective analysis of 1790 patients. However, only 46 subjects were in their endostapler cohort and therefore comparisons between methods is limited.

Other studies have highlighted the cost-effectiveness of EL [34,36]. In the included studies cost reporting was inconsistent, with some studies quoting cost per loop [21,23,24], rather than total operative cost. Some studies failed to report cost [17,18]. The majority of studies concluded that EL were a cost-effective method for securing the appendiceal stump. Compared with staples, Beldi et al. [15], Rakic et al. [16], and recently Kim et al. [25] found EL to be significantly cheaper than the endostapler method. The only study that compared EL with sutures found a decrease in operative cost of €380 euros per case when sutures were used [19].

Endoclips are easily applied laparoscopically and increase procedural efficiency, reducing both operative time and equipment cost [34]. The use of titanium and polymeric endoclips have both been described, however a direct comparison between these two types of clip when securing the appendiceal stump is yet to be performed.

The three randomized control trials included in this review had an average of 34.3 patients in each arm. Of these, only Ates et al. [31] performed a power calculation. The methods of randomization included computer generation and random allocation. However, there was no comment on the level of blinding employed in the allocated intervention in any of the trials.

Endoclips had one of the lowest reported rates of complications (7.1%) with a quicker mean operative time (47.7 min) and the most cost-effective method of stump closure at €29.73 per clip. Therefore endoclips are potentially the most efficient and cost-effective stump closure method. However, these results should be interpreted with caution due to limited patient numbers within the studies compared to other closure methods. There is a requirement for further well-designed blinded randomized control trials, with adequate power calculations.

In addition, the application of endoclips may be inhibited by appendix anatomy. It is generally accepted that the largest recommended diameter of stump closure that can be safely closed by polymeric clips is 10 mm [29]. However it is not uncommon for the base of the appendix to exceed this diameter, especially when acutely inflamed, and this may therefore limit the safe use of these devices. Although several studies did not report any problems with clip application or postoperative issues, Ates et al. did note that large clips (11 mm) were required for 2 patients in their groups, and that postoperative pain in one of the patients on postoperative day 3 was due to slippage of the titanium clip into the pelvic area. Additionally, one patient with a metallic clip in situ developed a post-operative abscess. These complications of slippage and abscess formation were also noted by Alis et al. [30]. They concluded that at least 1 cm of healthy tissue at the level of the appendiceal base is required for endoclip application, and if this is not present then an endostapler should be used. However, they did not specify if their study adhered to this as an influential criterion in their selected operative technique. Delibegovic et al. [20] noted that one of their patients was not eligible to be included in their Hem-o-lok sample due to the size of the bulging appendix base at operation being so large that the XL clip was not able to securely encircle the base of the appendix. However, their complication rates were low and comparable with their EL cohort. Gonenc et al. [28] describe a technique of twisting the appendix clockwise or counterclockwise, and applying a second clip parallel to the first in the opposite direction (cross-clipping) to secure the appendix stump when the appendix base exceeds the size of the clip. Rickert et al. [29] report they did not undertake stump closure with endoclips when the base of the appendix exceeded the size of the endoclip or when inflammation extended to the caecum due to safety concerns. They did not define ‘severe’ appendicitis or specify how broad the base would be in order to deem it unsafe for closure with clips. They reported this in four cases due to severe inflammation, with the stump being secured with an endostapler or Roeder loop as an alternative.

Another potential problem is clip migration. There have been several case reports describing the migration of titanium clips in cholecystectomy from their original position into the common bile duct [[37], [38], [39], [40]]. This has not been described for LA, and may be related to the diameter of the lumen of the large bowel. The diameter of the large bowel is much larger than that of the common bile duct, and so migration of clips is not usually an issue given that there is no associated significant occlusion, unless it this occurs acutely when the appendiceal stump remains unhealed.

Our findings are supported by a recent systematic review investigating the use of polymeric clips in stump closure. Knight et al. [41] noted that polymeric clips were the cheapest method (20.47 euros) of stump closure, and also had the lowest rate of complications (2.7%) when compared with other closure methods, with no adverse effect on operative time or duration of in-patient stay.

Mechanical staplers provide secure closure of the appendiceal stump, with easy handling and reduced intra-abdominal abscess rates, possibly from prevention of protruding mucosa in closure [34]. Mechanical endostaplers reduce the rate of colocutaneous fistula and enable the treatment of complicated appendicitis including the presence of necrosis, which is less effectively treated with EL or the application of clips [36].

Additionally, Beldi et al. [15] report a lower rate of surgical site infection and hospital re-admissions with an endostapler device compared to EL [12]. A meta-analysis combining the results of four randomized controlled trials between endostapler and EL closure determined that operative time and wound infection rates were lower in the endostapler group, with a reduction in post-operative ileus [9]. We report that intra-operative complication rates are among the lowest of all closure methods (3.56%). However, this was not the case for post-operative complication rates (8.34%). The cost of the stapler device is however prohibitive [42]. Figures range between €264.92 to €970.70 in the three studies which reported costs. Despite this the mechanical stapler remains one of the most common methods of appendiceal stump closure. 3878 patients were included in five large cohort studies, the majority prospective in nature. However none were randomized trials and therefore selection bias may exist within the studies. It is important to bear in mind that endostaplers are more likely to be used in the most severe cases of appendicitis, often in the presence of stump necrosis, and therefore complication rates associated with endostaplers may be falsely elevated. The longer in-patient stay (4.2 days) compared to other methods may also be explained by this principle, however operative time was comparable with other methods.

With regards to closure using laparoscopic sutures, complication rates were found to be high compared to other methods. However these figures were heavily influenced by the findings of Ates et al. [31]. No explanation was provided by the authors to account for the elevated complication rate, however a small sample size may have contributed to their findings.

It is also hypothesized that the mucosa can be everted when performing suture closure of the appendiceal stump, subsequently increasing the likelihood of intra-abdominal bacterial contamination [22]. Additionally, the presence of knots and suture material may produce a tendency to irritate the neighbouring mucosa, increasing post-operative inflammation compared with less reactive methods of closure. This is corroborated by our findings, demonstrating proportionally higher rates of stump leak and intra-abdominal abscess formation.

In addition, the mean operating time of 68.2 min was the longest overall, likely due to the increase in technical difficulty required to apply the suture to the appendiceal stump. Although cost could not be analyzed due the lack of reporting in the included studies, Kiudelis et al. state that sutures decrease the total cost of appendicectomy by €380 compared to EL [19]. Additionally, Gonenc et al. [28] estimated the cost of one silk suture at €1.76. This compares to Rickert et al. [29] estimating the cost of one titanium clip at €20 and others estimating the cost of a polymeric clip at €8.78 and an EL at €35.12 (21).

In relation to cost estimation, there is considerable variation in how each method has been assessed. Many studies calculated the cost of the entire operation without breaking down the cost of the particular method itself, while several studies calculated the cost of methods per pack rather than individually. There was additional variation in the currency used to cost closure tools.

Comparison between studies was difficult due to heterogenous patient selection and outcomes measured. In 3 studies, the exclusion criteria described may have skewed results, particularly in relation to complication rates, and thus introduced bias in reporting. Ates et al. excluded patients with perforated appendicitis from their study of suture vs. titanium endoclips. In the prospective randomized trial by Gonenc et al. comparing endoclips to sutures, patients with sepsis or septic shock on admission were excluded from their study, as were those diagnosed with complex appendicitis intraoperatively.

Importantly, none of the studies included in our review used any previously described grading system for appendicitis severity such as the Disease Severity Score [43], Appendicitis Inflammatory Response Score [44], or imaging severity scoring such as the CT-determined severity score [45], in order to further stratify their evaluation of ‘severe’ appendicitis. The only references to appendicitis severity influencing outcomes was from the study by Rickert et al., who deemed ‘severe’ appendicitis to preclude their sample from having the appendiceal stump being closed by clips.

The wide absence of such categorization of severity may well signify that there is a need to ensure that an accepted scoring system is used in future randomized studies.

Our findings are consistent with a recent Cochrane review which assessed all published randomized controlled trials assessing various methods of appendiceal stump closure up to 2017 [46]. They found no significant difference in the rate of overall complications, rate of intra- and post-operative complications, and no difference in overall hospital stay. Furthermore, as per our findings, there was observed to be a notable difference in operative duration, with a shorter operating time (9 min) with the use of mechanical devices compared with ligatures.

## Limitations

We note that there was not enough information provided in the randomized controlled trials assessed to reliably evaluate differences in cost, quality of life or pain.

Therefore, implications for future research, should provide an accurate costing analysis, while examining the three main methods of stump closure. EL, endoclip (both titanium and polymeric clips) and endostapler in an adequately powered age, sex and comorbidity matched multi-center randomized trial. Such a trial should assess operative duration, length of stay and cost analysis using a universal denominator, while also reporting post-operative complications. Future studies should additionally aim to stratify outcomes based on evaluation utilising a recognized severity scoring system for acute appendicitis. This will enable comparison between patient outcomes whilst taking in to account the severity of appendicitis that patients experienced. This will optimize study design and minimize bias in results obtained. Furthermore, future studies should stratify findings based on pathological severity, using a validated grading system such as that of Gomes et al. [47].

Finally, studies of EL performing accurate cost analysis are required, together with adequately controlled randomized control trials comparing this method to polymeric clips, as both these methods been found to have the most efficient operation with the most favourable outcomes.

## Provenance and peer review

Not commissioned, externally peer reviewed.

## Ethical reports

Ethical approval not required (systematic review)

## Sources of funding

None.

## Author contribution

Navnit Makaram – Conception, data curation, analysis, manuscript preparation.

Stephen Knight – Data curation, statistical analysis, manuscript preparation.

Abdulla Ibrahim – Manuscript preparation.

Michael Wilson - Conception, Manuscript preparation, Pradeep Patil – Conception, Manuscript preparation.

## Trials register

1.Name of the registry: Not required (systematic review registered with PROSPERO)

2.Unique Identifying number or registration ID: PROSPERO registration number: CRD42019153567

3.Hyperlink to your specific registration (must be publicly accessible and will be checked): https://www.crd.york.ac.uk/prospero/#searchadvanced

## Guarantor

Nav0nit Makaram.

## Declaration of competing interest

No conflicts of interest.

## References

[1] Nshuti R., Kruger D., Luvhengo T.E. (2014). Clinical presentation of acute appendicitis in adults at the Chris Hani Baragwanath academic hospital. Int. J. Emerg. Med..

[2] Simpson J., Samaraweera A.P., Sara R.K. (2008). Acute appendicitis--a benign disease?. Ann. R. Coll. Surg. Engl..

[3] Jaschinski T., Mosch C.G., Eikermann M. (2018). Laparoscopic versus open surgery for suspected appendicitis. Cochrane Database Syst. Rev..

[4] Gorenoi V., Dintsios C.-M., Schönermark M.P. (2007). Laparoscopic vs. open appendectomy: systematic review of medical efficacy and health economic analysis. GMS Health Technol. Assess..

[5] Krisher S.L., Browne A., Dibbins A. (1960). Intra-abdominal abscess after laparoscopic appendectomy for perforated appendicitis. Archives of surg (Chicago, Ill).

[6] Sporn E., Petroski G.F., Mancini G.J. (2009). Laparoscopic appendectomy--is it worth the cost? Trend analysis in the US from 2000 to 2005. J. Am. Coll. Surg..

[7] Kockerling F., Schug-Pass C., Grund S. (2009). [Laparoscopic appendectomy. The new standard?. Der Chirurg; Zeitschrift fur alle Gebiete der Operativen Medizen.

[8] Rickert A., Krüger C.M., Runkel N. (2015). The TICAP-Study (titanium clips for appendicular stump closure): a prospective multicentre observational study on appendicular stump closure with an innovative titanium clip. BMC Surg..

[9] Kazemier G., in't Hof K.H., Saad S. (2006). Securing the appendiceal stump in laparoscopic appendectomy: evidence for routine stapling?. Surg. Endosc..

[10] Sajid M.S., Rimple J., Cheek E. (2009). Use of endo-GIA versus endo-loop for securing the appendicular stump in laparoscopic appendicectomy: a systematic review. Surg. Laparosc. Endosc. Percutaneous Tech..

[11] Wilson M.S.J., Knight S.R., Vaughan-Shaw P. (2018). Securing the appendiceal stump during emergency appendicectomy: options and influencing factors in current UK surgical practice. Surg. Laparosc. Endosc. Percutaneous Tech..

[12] Moher D., Liberati A., Tetzlaff J. (2009). Preferred reporting items for systematic reviews and meta-analyses: the PRISMA statement. PLoS Med..

[13] oanda Average exchange rates. https://www.oanda.com/currency/average.

[14] Team R.C.R. (2013). A Language and Environment for Statistical Computing.

[15] Beldi G., Vorburger S.A., Bruegger L.E. (2006). Analysis of stapling versus endoloops in appendiceal stump closure. Br. J. Surg..

[16] Rakic M., Jukic M., Pogorelic Z. (2014). Analysis of endoloops and endostaples for closing the appendiceal stump during laparoscopic appendectomy. Surg. Today.

[17] Swank H.A., van Rossem C.C., van Geloven A.A. (2014). Endostapler or endoloops for securing the appendiceal stump in laparoscopic appendectomy: a retrospective cohort study. Surg. Endosc..

[18] Sahm M., Kube R., Schmidt S. (2011). Current analysis of endoloops in appendiceal stump closure. Surg. Endosc..

[19] Kiudelis M., Ignatavicius P., Zviniene K. (2013). Analysis of intracorporeal knotting with invaginating suture versus endoloops in appendiceal stump closure. Wideochir Inne Tech Maloinwazyjne.

[20] Delibegovic S., Matovic E. (2009). Hem-o-lok plastic clips in securing of the base of the appendix during laparoscopic appendectomy. Surg. Endosc..

[21] Colak E., Kement M., Ozlem N. (2013). A comparison of nonabsorbable polymeric clips and endoloop ligatures for the closure of the appendicular stump in laparoscopic appendectomy: a prospective, randomized study. Surg. Laparosc. Endosc. Percutaneous Tech..

[22] Jenwitheesuk K., Chotikawanich E., Saeseow O.T. (2012). Laparoscopic appendectomy: results of a new technique for stump management. J. Med. Assoc. Thailand = Chotmaihet Thangphaet.

[23] Lucchi A., Berti P., Grassia M. (2017). Laparoscopic appendectomy: hem-o-lok versus Endoloop in stump closure. Updates in Surg.

[24] Wilson M., Maniam P., Ibrahim A. (2018). Polymeric clips are a quicker and cheaper alternative to endoscopic ligatures for securing the appendiceal stump during laparoscopic appendicectomy. Ann. R. Coll. Surg. Engl..

[25] Kim S., Weireter L. (2018). Cost effectiveness of different methods of appendiceal stump closure during laparoscopic appendectomy. Am. Surg..

[26] Hanssen A., Plotnikov S., Dubois R. (2007). Laparoscopic appendectomy using a polymeric clip to close the appendicular stump. J. Soc. Laparoendosc. Surg: J. Soc. Laparoendosc. Surg..

[27] Strzalka M., Matyja M., Rembiasz K. (2014). Results of laparoscopic appendectomies performed with the use of titanium clips for closure of the appendicular stump. Pol. Przegl. Chir..

[28] Gonenc M., Gemici E., Kalayci M.U. (2012). Intracorporeal knotting versus metal endoclip application for the closure of the appendiceal stump during laparoscopic appendectomy in uncomplicated appendicitis. J. Laparoendosc. Adv. Surg. Tech. Part A.

[29] Rickert A., Bonninghoff R., Post S. (2012). Appendix stump closure with titanium clips in laparoscopic appendectomy. Langenbeck's Arch. Surg..

[30] Alis H., Gonenc M., Deniztas C. (2012). Metal endoclips for the closure of the appendiceal stump in laparoscopic appendectomy. Tech. Coloproctol..

[31] Ates M., Dirican A., Ince V. (2012). Comparison of intracorporeal knot-tying suture (polyglactin) and titanium endoclips in laparoscopic appendiceal stump closure: a prospective randomized study. Surg. Laparosc. Endosc. Percutaneous Tech..

[32] Al-Temimi M.H., Berglin M.A., Kim E.G. (2017). Endostapler versus Hem-O-Lok clip to secure the appendiceal stump and mesoappendix during laparoscopic appendectomy. Am. J. Surg..

[33] Kliuchanok K., Keßler W., Partecke I. (2019). A comparison of non-absorbable polymeric clips and staplers for laparoscopic appendiceal stump closure: analysis of 618 adult patients. Langenbeck's Arch. Surg..

[34] Shaikh F.M., Bajwa R., McDonnell C.O. (2015). Management of appendiceal stump in laparoscopic appendectomy--clips or ligature: a systematic review and meta-analysis. J. Laparoendosc. Adv. Surg. Tech. Part A.

[35] Cristalli B.G., Izard V., Jacob D. (1991). Laparoscopic appendectomy using a clip applier. Surg. Endosc..

[36] Gomes C.A., Nunes T.A., Soares C. (2012). The appendiceal stump closure during laparoscopy: historical, surgical, and future perspectives. Surg. Laparosc. Endosc. Percutaneous Tech..

[37] Matsumoto H., Ikeda E., Mitsunaga S. (2000). Choledochal stenosis and lithiasis caused by penetration and migration of surgical metal clips. J. Hepato-Biliary-Pancreatic Surg..

[38] Tsumura H., Ichikawa T., Kagawa T. (2002). Failure of endoscopic removal of common bile duct stones due to endo-clip migration following laparoscopic cholecystectomy. J. Hepato-Biliary-Pancreatic Surg..

[39] Chong V.H., Chong C.F. (2010). Biliary complications secondary to post-cholecystectomy clip migration: a review of 69 cases. J. Gastrointest. Surg: Offc. J. Soc. Surg. Alimentary Tract.

[40] Ng W.T., Kong C.K., Lee W.M. (1999). Migration of three endoclips following laparoscopic cholecystectomy. J. R. Coll. Surg. Edinb..

[41] Knight S.R., Ibrahim A., Makaram N. (2019). The use of polymeric clips in securing the appendiceal stump during laparoscopic appendicectomy: a systematic review. Eur. J. Trauma Emerg. Surg. Offc. Pub. Eur. Trauma. Soc..

[42] Vettoretto N., Agresta F. (2011). A brief review of laparoscopic appendectomy: the issues and the evidence. Tech. Coloproctol..

[43] Garst G.C., Moore E.E., Banerjee M.N. (2013). Acute appendicitis: a disease severity score for the acute care surgeon. J. Trauma. Acute. Care. Surg.

[44] Andersson M., Andersson R.E. (2008). The appendicitis inflammatory response score: a tool for the diagnosis of acute appendicitis that outperforms the Alvarado score. World J. Surg..

[45] Kim H.C., Yang D.M., Lee C.M. (2011). Acute appendicitis: relationships between CT-determined severities and serum white blood cell counts and C-reactive protein levels. Br. J. Radiol..

[46] Mannu G.S., Sudul M.K., Bettencourt‐Silva J.H. (2017). Closure methods of the appendix stump for complications during laparoscopic appendectomy. Cochrane Database Syst. Rev..

[47] Gomes C.A., Nunes T.A., Fonseca Chebli J.M. (2012). Laparoscopy grading system of acute appendicitis: new insight for future trials. Surg. Laparosc. Endosc. Percutaneous Tech..

